# Elevated serum levels of bone sialoprotein during ICU treatment predict long-term mortality in critically ill patients

**DOI:** 10.1038/s41598-018-28201-7

**Published:** 2018-06-27

**Authors:** Mark Luedde, Sanchari Roy, Hans-Joerg Hippe, David Vargas Cardenas, Martina Spehlmann, Mihael Vucur, Pia Hoening, Sven Loosen, Norbert Frey, Christian Trautwein, Tom Luedde, Alexander Koch, Frank Tacke, Christoph Roderburg

**Affiliations:** 1 Department of Internal Medicine III, University Hospital of Schleswig Holstein, Campus Kiel, Rosalind-Franklin-Str. 12, 24105 Kiel, Germany; 2 Department of Medicine III, University Hospital RWTH Aachen, Pauwelsstrasse 30, 52074 Aachen, Germany

## Abstract

Bone sialoprotein (BSP), a member of the SIBLINGs (for Small Integrin-Binding LIgand, N-linked Glycoproteins) family, has recently be associated to inflammatory and infectious diseases. We therefore measured BSP concentrations in 136 patients at admission to the intensive care unit (ICU) and 3 days of ICU. BSP levels were compared to 36 healthy blood donors and correlated to clinical data. In these analysis, BSP serum levels were strongly elevated at the time point of admission to the ICU when compared to healthy controls. Moreover BSP concentrations were significantly elevated after 3 days of treatment on the intensive care unit. A further increase in BSP levels was detected in patients with higher APACHE-II-scores and in patients with septic disease. While in most patients, BSP levels decreased during the first three days of treatment on a medical ICU, patients with persistently elevated BSP levels displayed an unfavorable outcome. In these patients, persistently elevated BSP concentrations were a superior predictor of mortality than established indicators of patient´ prognosis such as the SAPS2 or the APACHE-II score. In summary, our data argue for a novel utility for BSP as a biomarker in patients treated on a medical ICU.

## Introduction

Despite intensive research, pharmacological treatment strategies for sepsis still rely on the early initiation of antibiotic and supportive treatment. Serum based markers allowing the early diagnosis of septic disease or predicting the clinical fate of critically ill patients would be of high relevance for guiding patients’ treatment, highlighting the persistent demand for new biomarkers in the context of critical illness and sepsis^1^.

The so called Small integrin-binding ligand N-linked glycoproteins (SIBLINGs)- family consists of five integrin-binding glycophosphoproteins, namely the bone sialoprotein (BSP), dentin matrix protein 1 (DMP1), dentin sialophosphoprotein (DSPP), matrix extracellular phosphoglycoprotein (MEPE) and osteopontin (OPN). SIBLINGs are soluble, secreted proteins which are involved in the regulation of different biological processes such as cell proliferation, apoptosis, angiogenesis, wound repair, and regulation of ECM remodeling. On a molecular level, SIBLINGs have been shown to modulate various signalling pathways with a critical role in inflammation and infection, including PI3K/Akt-, the Ras/Raf- and NF-κB- pathway. SIBLINGs have been suggested to be involved in the pathophysiology of various benign and malignant diseases. Elevated expression and serum concentrations of Osteopontin, the most widely studied member of the SIBLINGs family, were shown in samples from patients sufferering from systemic or focal inflammation, occurring e.g. during tuberculosis^2^, multiple sclerosis^3^, lupus erythematosus^4^ and Crohn’s disease^5^, highlighting the role of this protein family in inflammatory and infectious diseases. We have recently reported that serum levels of Osteopontin, another SIBLING protein, were significantly elevated in critically ill patients. Of note, elevated OPN levels were strong predictor for an unfavourable patients’ outcome^6^. Moreover, we demonstrated that in patients with cholangiocellur carcinoma, display higher serum Osteopontin concentrations when compared to controls. In these patients, elevated serum levels of Osteopontin indicated a poor patients’ outcome and more postoperative complications^7^.

Recently, a function of members of the SIBLINGs family was suggested in the regulation of inflammation and immunity, it is presently unknown if bone sialoprotein plays a functional role in systemic infections in ICU patients or if BSP might have a function as a biomarker in critically ill patients. To address this question we measured BSP concentrations in the serum of critically ill patients that were treated on a large medical ICU within a tertiary referral center. We specifically analyzed whether BSP levels were elevated in patients treated on the ICU and subsequently might have a diagnostic value in the context of sepsis or multi-organ failure. Finally, we analyzed whether BSP measurements might serve marker indicating short- and long-term outcome in critical illness and sepsis.

## Materials and Methods

### Study design and patient characteristics

We enrolled 136 patients that were admitted our intensive care unit at the University Hospital (RWTH) Aachen (Table 1). 36 healthy blood donors served as controls. The study protocol was approved by the local ethics committee and conducted in accordance with the ethical standards laid down in the Declaration of Helsinki (ethics committee of the University Hospital Aachen, RWTH-University, Aachen, Germany, reference number EK 150/06). Written informed consent was obtained from the patient, his or her spouse or the appointed legal guardian. Presence of sepsis was considered if the criteria defined within the sepsis-3 criteria for sepsis, severe sepsis and septic shock were fulfilled. Otherwise, patients were considered as non-sepsis patients^8,9^. Obesity was assumed in patients with a body mass index of >30 kg/m² at ICU admission (prior to any treatment such as fluid resuscitation), type 2 diabetes in patients with a respective medical history and a concomitant intake of diabetes related medication.

**Table 1:** Baseline patient characteristics.

Parameter	all patients
Number	136
Sex (male/female)	76/60
Age median (range)[years]	66 (18–90)
Sepsis (yes/no)	96/40
APACHE-II score median d1 (range)	18.5 (3–40)
APACHE-II score median d3 (range)	22 (2–36)
SAPS2 score median d1 (range)	44 (9–80)
SAPS2 score median d3 (range)	41 (8–69)
SOFA score median d1 (range)	10 (0–17)
SOFA score median d3 (range)	10 (0–18)
ICU days median (range)	9 (1–137)
Death during ICU (%)	25.7
Death during ICU or follow-up (%)	48.1
Body mass index median (range)	26.12 (15.9–59.5)
Creatinine median (range) [mg/dl]	1.5 (0.1–21.6)
Albumin median (range) [g/l]	27.1 (11–44)
WBC median (range) [×10³/µl]	12.7 (0–208)
CRP median (range) [mg/dl]	122.5 (0–230)
Procalcitonin median (range) [µg/l]	1.0 (0–125)
Interleukin-6 median (range) [pg/ml]	110 (0–26000)
BSP at admission median [ng/ml]	38.43 (0.1–586.81)
BSP at day 3 median [ng/ml]	28.11 (0.1–457.41)

APACHE, Acute Physiology and Chronic Health Evaluation; CRP, C-reactive protein; ICU, intensive care unit; SAPS, simplified acute physiology score; WBC, white blood cell count.

### Determination and definitions of relevant parameters in critically ill patients

Interleukin-6 (IL-6), Interleukin-10 (IL-10), TNF, procalcitonin (PCT), soluble urokinase plasminogen activator receptor (suPAR), NTproCNP, Ghrelin, Hyaluronic acid and A proliferation inducing ligand (APRIL) were measured as described previously^10–16^. Glomerular filtration rates (GFR) were calculated on basis of serum cystatin C levels. ICU mortality was defined as death on ICU; overall mortality included death at the ICU or during the observation period (after discharge from ICU and hospital;^10–16^).

### Determination of bone sialoprotein serum concentrations by ELISA

BSP serum concentrations were analyzed using a commercial enzyme immunoassay according to the manufacturers’ instructions (Human BSP ELISA, Hölzel Diagnostika Handels GmbH, Cat. Nr. abx575181).

### Statistical analysis

All statistical analyses were performed with SPSS (SPSS, Chicago, IL, USA) or GraphPad Prism 5.0 as previously described^12,17^. Optimal cut off points to differentiate between two groups were calculated using the Youden Index, as described elsewhere^18^.

## Results

### Critically ill patients demonstrate elevated BSP serum levels at admission to the ICU

**Figure 1:**
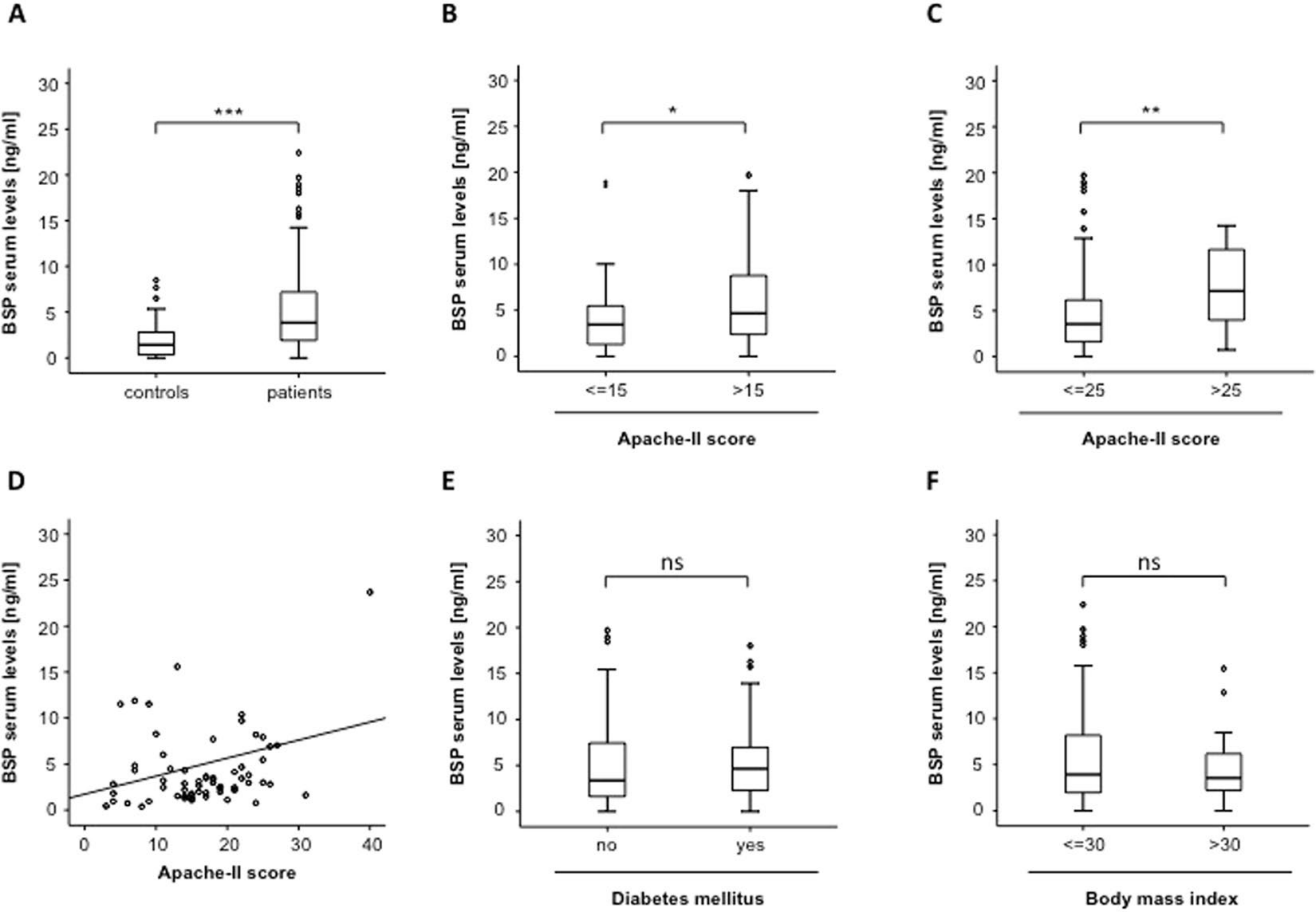
Critically ill patients demonstrate elevated BSP serum levels at admission to the ICU. **(A)** BSP serum levels at admission to the ICU were significantly elevated in patients with critical illness (n = 136) compared to healthy blood donors (n = 50). **(B**,**C)** BSP concentrations at admission to the ICU were further elevated in patients with more severe disease state according to initial APACHE-II scores (cut-offs are given in the figure). **(D)** Serum BSP concentrations were significantly correlated with the disease severity according to APACHE-II score in in critically ill patients. **(E**,**F)** Serum BSP levels in critically ill patients were independent on the presence of obesity (BMI > 30 kg/m^2^) or the presence of diabetes mellitus type 2. N values are given in the figures and tables; Spearman rank correlation test, correlation coefficient r, and P-values are given. *p < 0.05, **p < 0.01, ***p < 0.001.

To examine a potential role of BSP serum concentrations as a biomarker in patients treated on a medical intensive care unit, we analyzed BSP serum concentrations critically ill patients at admission to the ICU. In this analysis, BSP levels were significantly higher in patients, when compared to healthy blood donors. We next analyzed the impact of disease severity on BSP levels in patients with critical illness. Therefore patients were subdivided into those with low APACHE-II-scores and those with high APACHE-II-scores, respectively. Of note, patients with high APACHE-II-scores (>15) revealed higher BSP serum levels, when compared to patients less severe disease states (Fig. 1B) and this observation remained consistent when different cut-offs were analyzed (Fig. 1C, Supplementary Figure 1). In line, BSP serum levels significantly correlated with the APACHE-II score (Fig. 1D, Table 2).

**Table 2:** Correlations of bone sialoprotein serum concentrations with other laboratory markers. NA, not assessed.

	BSP at admission vs. laboratory markers at admission day		BSP at day 3 vs. laboratory markers at admission day	
	r	P	r	p
**Markers of inflammation**				
CRP	0.258	0.003	0.228	0.012
Procalcitonin	0.377	0.001	0.425	0.094
IL-10	0.358	0.008	0.159	0.254
IL-6	0.325	0.001	0.262	0.033
**Markers of organ function**				
Creatinine	0.067	0.439	0.124	0.239
GFR	0.004	0.970	−0.197	0.113
AST	0.115	0.210	−0.194	0.075
ALT	0.123	0.156	−0.314	0.002
GLDH	0.100	0.293	−0.066	0.668
**Bilirubin total**	−0.013	0.883	−0.092	0.382
GGT	−0.005	0.957	−0.113	0.210
Albumin	0.005	0.965	−0.110	0.364
Iono lactat	0.225	0.012	0.101	0.362
Base excess	−0.288	0.001	0.143	0.193
pH	−0.178	0.047	−0.089	0.420
BNP	0.268	0.044	0.369	0.010
**Clinical scoring**				
Apache II	0.255	0.009	0.231	0.069
SAPS2	0.698	0.052	0.318	0.014
SOFA	0.296	0.051	0.678	0.045
**Experimental sepsis markers**				
APRIL	0.263	0.012	0.014	0.914
NTproCNP	0.013	0.884	0.229	0.028
Osteopontin	0.078	0.509	0.076	0.675
Leptin	−0.080	0.539	0.130	0.324
Leptin receptor	0.138	0.292	−0.107	0.419
Adiponectin	0.021	0.875	0.013	0.920
Ghrelin	0.077	0.556	−0.180	0.168
Resistin	0.367	0.004	0.037	0.780
**Other**				
Fibrinogen	0.223	0.016	0.068	0.547
INR	0.193	0.025	0.189	0.072
Phosphate	−0.020	0.827	0.093	0.396

Different metabolic diseases including type 2 diabetes, hyperinsulinemia and obesity were recently linked with elevaed serum levels of osteoblast related proteins including BSP. To analyze the impact of metabolic diseases on BSP serum levels in critically ill patients, we analyzed BSP levels with respect to the presence of type 2 diabetes and. In these analysis BSP levels at admission to the intensive care unit were almost identical in patients with or without preexisting diabetes or obesity (Fig. 1E,F). Moreover, serum BSP concentrations did not correlate with the patients’ body mass index (BMI) or markers for a deregulated metabolic state (serum levels of Adiponectin, Leptin, leptin receptor) or insulin resistance (serum levels of Insulin, C-peptide, HbA1c, HOMA; Table 2). In addition, BSP concentrations did not vary with respect to patients’ age or gender (not shown).

**Figure 2:**
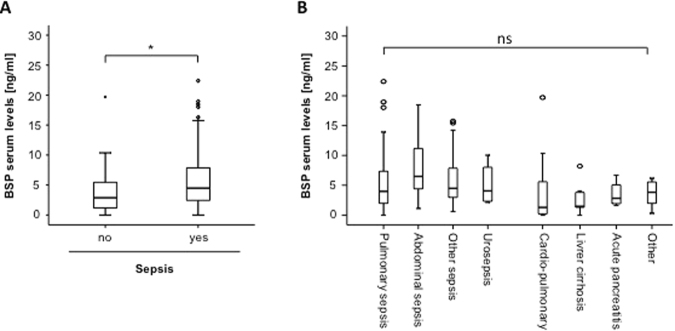
Serum BSP levels are elevated in sepsis. **(A)** Critically ill patients with sepsis (n = 96) displayed significantly higher BSP serum concentrations compared to patients without sepsis (n = 40, U-test). **(B)** BSP serum concentrations were not different in patients with different etiologies of critical illness. *p < 0.05.

We recently demonstrated that OPN might be used as an indicator for septic disease in patients with critical illness treated on a medical ICU^6^. Based on these recent data we examined the impact of sepsis on serum BSP levels in our cohort of patients. Interestingly, patients with septic etiology of critical illness demonstrated significantly higher BSP serum levels compared to patients with other disease etiology (Fig. 2A). Our cohort consisted of 96 patients fulfilling the criteria of sepsis, and 40 that did not (Table 1)^19,20^. Within the sepsis cohort, most patients suffered from pneumonia, while within the non-septic group of patients cardiovascular diseases represented the predominant etiology of critical illness. Notably, we found only minor differences between the different etiologies of septic disease (Fig. 2B), highlighting that BSP serum levels are elevated in septic patients regardless of the diseases etiology.

### Development of BSP serum levels during the first 72 hours of treatment on the ICU

**Figure 3:**
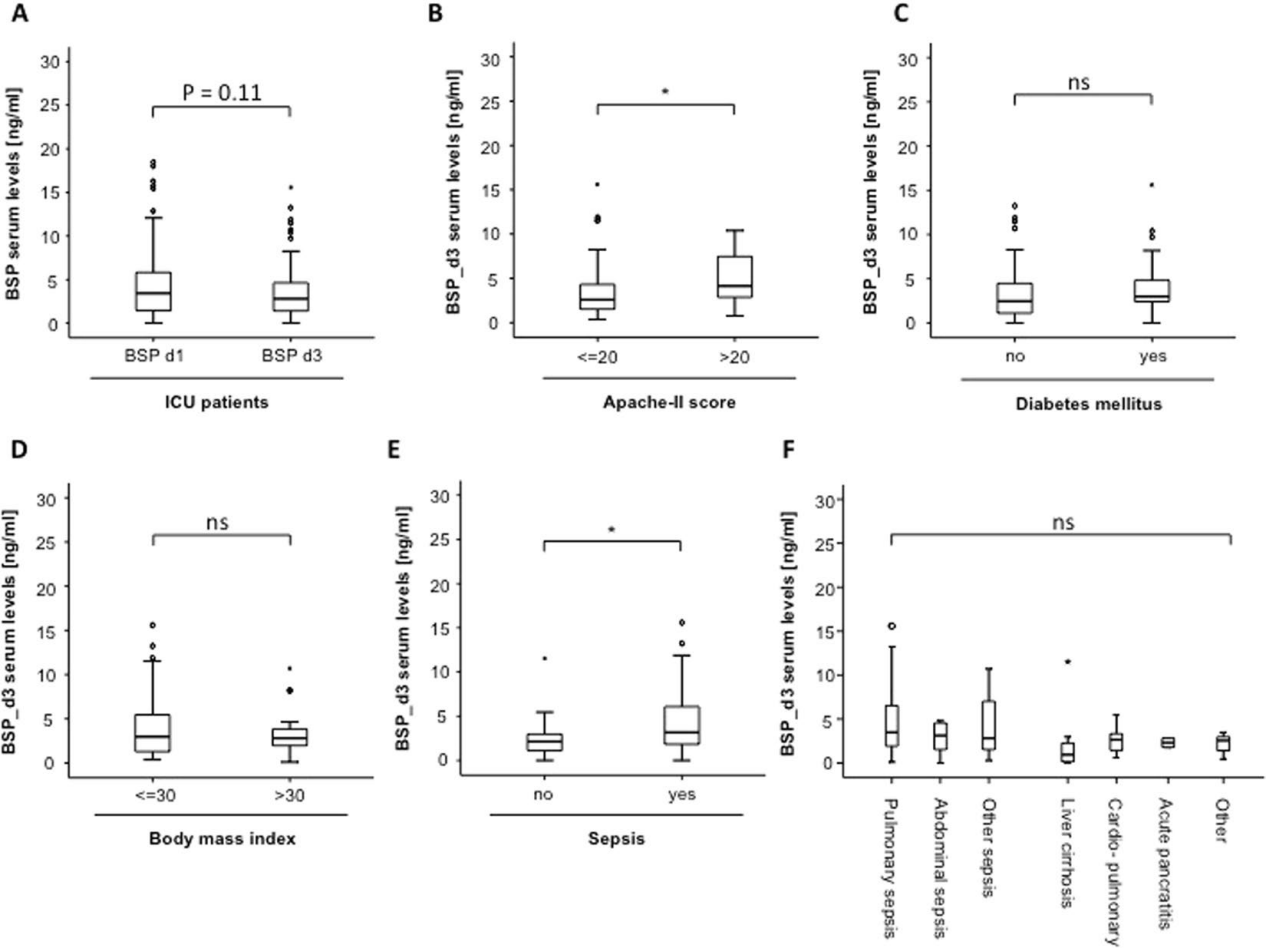
Development of BSP serum levels during the first 72 hours of treatment on the ICU. **(A)** Serum BSP concentrations demonstrated a slight decrease during the first 3 days of ICU treatment (Wilcoxon signed-rank test was used to assess the statistical difference between the repeated measurements in the same patient). **(B)** Serum BSP concentrations at d3 were compared between patients with different disease severities according to their respective APACHE-II-scores. **(C)** Serum BSP concentrations at day 3 were compared between patients with septic disease and patients with a non-septic disease etiology. **(D)** BSP serum concentrations after 3 days of ICU treatment were independent of the specific disease etiology. **(E**,**F)** Serum BSP levels after 3 days of ICU treatment were independent on the presence of obesity or diabetes mellitus type 2 in our cohort of patients. *p < 0.05.

We measured BSP serum concentration after 72 hours of ICU treatment in our cohort of patients. Similar to elevated BSP levels at admission, serum BSP concentrations were significantly elevated at this later time-point (Fig. 3A) and demonstrated a trend towards further increased levels in patients with more severe disease (Fig. 3B). While BSP levels after 3 days of ICU stay were similar in patients with our without diabetes or obesity respectively, (Fig. 3C,D), we found a further increase in BSP concentrations when sepsis was present compared to patients with other disease etiology (Fig. 3E). We next repeated the subgroup analysis presented in Fig. 1F to detect the impact of different disease etiologies on BSP serum concentrations at day 3. Similar to the results obtained at admission to the ICU, BSP serum levels at day 3 were similar in all analyzed subgroups.

### Association of elevated BSP concentrations and patients´ ICU or overall survival

**Figure 4:**
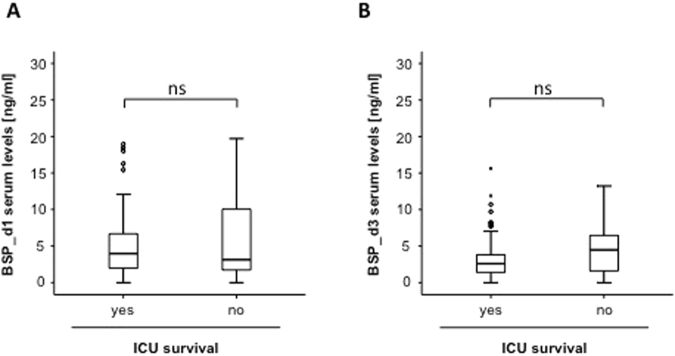
Association of elevated BSP concentrations and patients’ ICU survival. **(A)** BSP serum levels at admission did not differ between patients that died during their ICU treatment and survivors. **(B)** BSP levels at day 3 did not differ between patients that died during their ICU treatment and survivors.

We next attempted to analyze whether BSP measurements are suitable to predict mortality in patients with critical illness. Therefore we first measured BSP concentrations ad admission in patients that succumbed to death during ICU treatment and those patients that survived. Interestingly, in this analysis median BSP concentrations within the group of the survivors and patients that died were almost identical (Fig. 4A). Next, we compared BSP serum concentrations after three days of ICU treatment in patients that did not survive their ICU-stay to those from survivors. Despite a strong trend towards lower levels in surviving patients became apparent, the difference again did not reach statistical significance (p = 0.113; Fig. 4B).

**Figure 5:**
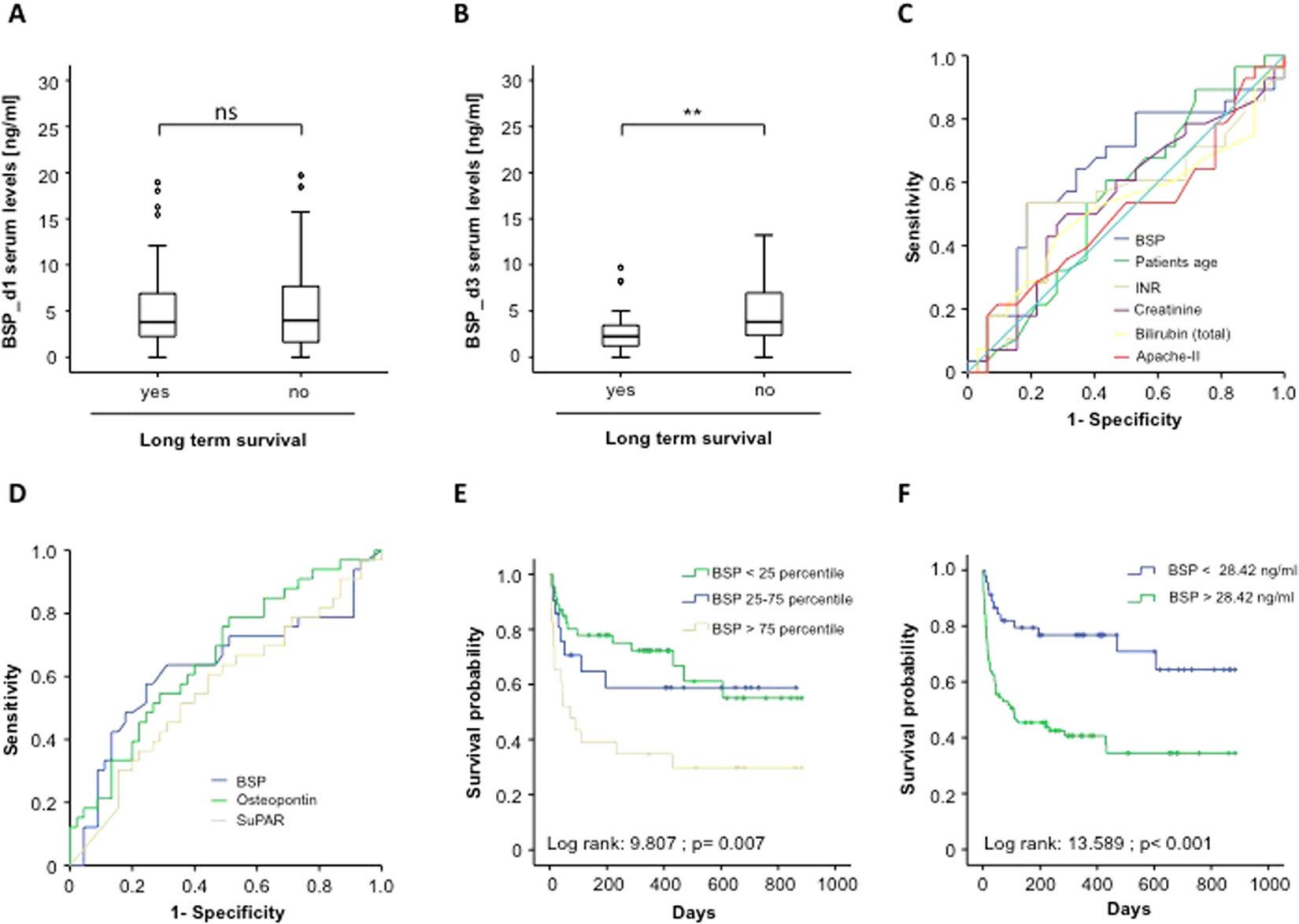
Association of elevated BSP concentrations and patients’ overall survival. **(A)** Serum concentrations of BSP at admission to the ICU are displayed with respect to patients’ overall survival. **(B)** Serum concentrations of BSP at d3 are displayed with respect to patients’ overall survival. **(C)** ROC curve analyses comparing the prognostic value of BSP levels at day 3 for overall survival with that of patients’ age (AUC 0.554), INR (AUC 0.550), creatinine (AUC 0.548), bilirubine (AUC 0.520) and the APACHE-II–score (AUC 0.504). **(D)** ROC curve analyses comparing the prognostic value of BSP levels at day 3 for overall survival (AUC 0.630) with that of Osteopontin (AUC 0.661) or suPAR (AUC 0.565). **(E**,**F)** Kaplan-Meier survival curves analysis.

We next compared BSP serum levels between patients that dies during the long term follow up and patients that survived. Notably, no difference in BSP concentrations at admission between patients succumbed to death during the long term follow-up and survivors was observed. In contrast, serum BSP levels after 72 hours of treatment were significantly higher levels in patients that died during long-term observation when compared to patients that survived (Fig. 5A,B). To compare the prognostic value of serum BSP levels to that of other markers routinely assessed in the context of ICU treatment, we next performed ROC curve analysis. Interestingly, despite the prognostic power of all analyzed markers for predicting long-term mortality was rather low, BSP was still superior to that of patients’ age, INR, serum creatinine or bilirubin concentrations and the APACHE-II-score (Fig. 5C). Moreover, BSP was superior to measurements of serum osteopontin concentrations and almost equal to suPAR, representing experimental markers of inflammation and sepsis (Fig. 5D).

Based on these results highlighting a prognostic value of serum BSP concentrations, Kaplan Meier curve analysis and cox regression analysis was done to determine the impact of elevated BSP levels on patient survival in our cohort of ICU- patients. Of note, patients with elevated BSP concentrations (4th quartile) displayed a significantly higher mortality compared to the other patients (Fig. 5E). We determined the optimal threshold for prediction of patients’ prognosis by using the Youden-Index. Based on a BSP value of 28.42 ng/ml, which best fulfilled these requirements, we performed Kaplan-Meier survival analysis, showing that patients with high BSP serum levels after 72 hours of treatment displayed an unfavorable outcome when compared to patients with lower values (Fig. 5F).

### Correlation of BSP levels to parameters routinely accessed in ICU patients

**Figure 6:**
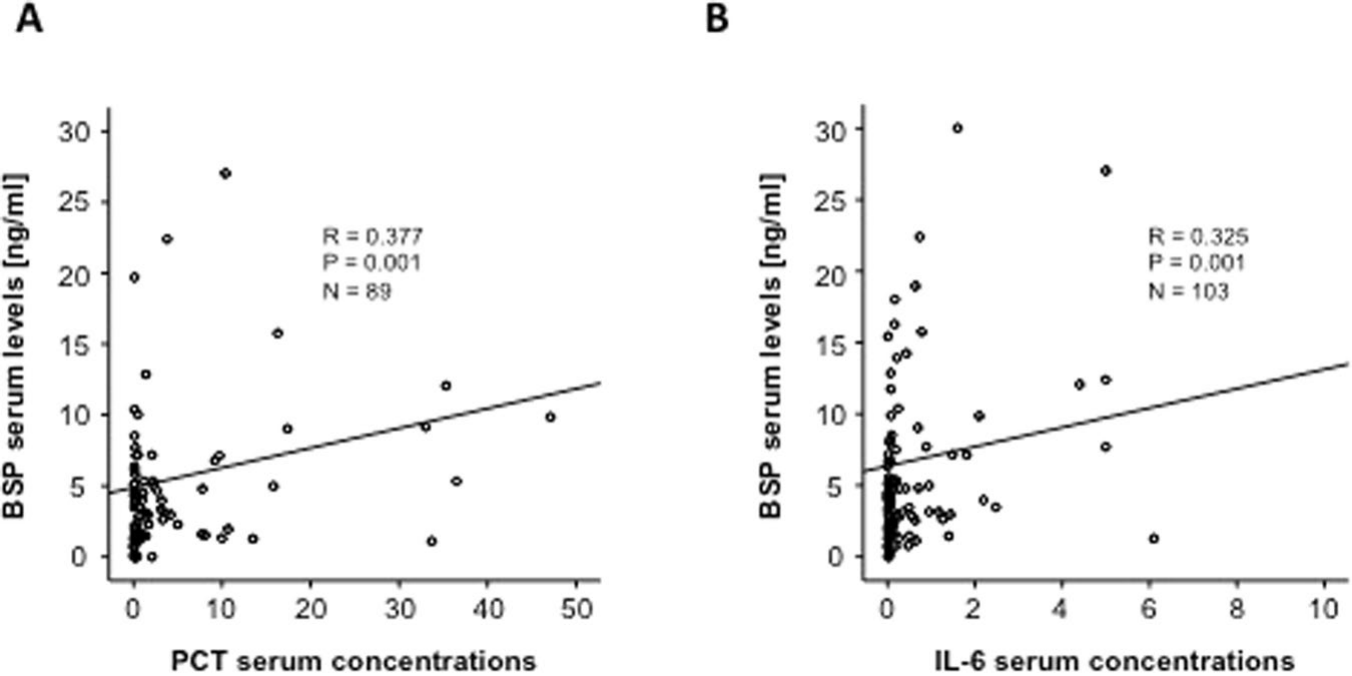
SBP serum concentrations are correlated with markers of systemic inflammation. Serum BSP concentrations in ICU patients are correlated with PCT or IL-6. Spearman rank correlation test, correlation coefficient r, and P-values are given.

We next performed correlation analysis with a broad panel of laboratory parameters. These analyses demonstrated that BSP levels were strongly correlated with markers indicating the presence of systemic infection and bacterial infection. As such high BSP levels were correlated to CRP (d1: r = 0.258, p = 0.003; d3: r = 0.228, p = 0.012), PCT (d1: r = 0.377, p < 0.001; d3: r = 0.425, p = 0.094, Fig. 6), IL-6 (d1: r = 0.325, p = 0.001; d3: r = 0.262, p = 0.033, Fig. 6A,B, Table 2) and IL-10 (d1: r = 0.358, p = 0.008; d3: r = 0.159, p = 0.254). Interestingly, no correlations to parameters used in the assessment of organ failure were found (Table 2). Nevertheless, high BSP correlated to markers for an unfavorable prognosis such as ventilation settings, base excess (d1: r = −0.288, p = 0.001, d3: r = −0.143, p = 0.193) and serum lactate levels (d1: r = 0.225, p = 0.012, d3: r = 0.362, p = 0.101). Consequently, BSP serum concentrations correlated with the APACHE II-, the sequential organ failure assessment (SOFA)- and the SAPS2-scores (Table 2). In line to this interesting finding we found a strong correlation between BSP serum concentrations and other experimental markers such as APRIL or Resistin (Table 3). Previously, direct link between serum concentrations of Osteopontin and heart failure was demonstrated^21,22^. Moreover, brain natriuretic peptide (BNP) and Osteopontin concentrations were found to be directly correlated in critical illness^6^. Notably levels of BSP, another member of the SIBLINGS family, also correlated to BNP (d1: r = 0.268, p = 0.044; d3: r = 0.369, p = 0.010) in this cohort of ICU-patients.

**Table 3:** Disease etiology of the study population.

	sepsis	non-sepsis
	96	40
Etiology of sepsis critical illness Site of infection n (%)		
Pulmonary	55	
Abdominal	12	
Urogenital	6	
Other	23	
Etiology of non-sepsis critical illness n (%)		
cardiopulmonary disease		15
decompensated liver cirrhosis		9
acute pancreatitis		4
major bleeding		1
acute liver failure		1
non-sepsis other		10

## Discussion and Conclusions

We used a well characterized cohort of critically ill patients^23–26^ to demonstrate that BSP serum concentrations reflect a significant prognostic information, especially at day three after admission. These data not only demonstrate a previously unrecognized function of BSP as a biomarker in critical illness and sepsis, but also support a clinical relevance of bone sialoprotein in the pathogenesis of inflammatory diseases.

BSP is a major structural protein of the bone matrix. It constitutes approximately 12% of the non-collagenous proteins in human bone and is synthesized by skeletal-associated cell types, including hypertrophic chondrocytes, osteoblasts, osteocytes, and osteoclasts. The only extraskeletal site of its synthesis is the trophoblast^27^. BSP is a member of the SIBLING (Small Integrin-Binding LIgand, N-linked Glycoprotein) family of genetically related proteins that are clustered on human chromosome 4^28^. Other members of this family are dentin matrix protein 1 (DMP1), osteopontin, dentin-sialophosphoprotein (DSPP), statherin and Matrix Extracellular PhosphogloprotEin (MEPE)^29^. A unifying feature of the SIBLING proteins is an Acidic Serine Aspartate Rich MEPE associated motif (ASARM). This motif and the released ASARM peptide unfold their action regulating the activity of two proteins: FGF 23 and PHEX, playing important roles in mineralization, bone turnover, mechanotransduction, phosphate regulation and energy metabolism^30^. Besides these physiological functions, it has also been demonstrated, that BSP play an important role in bone mineralization and the development of bone cancer^31^. Moreover, BSP is involved into breast cancer cell adhesion to the bone^32^ and is also a predictive marker of bone metastases in resectable non-small-cell lung cancer^33^. While a functional importance of BSP in cancer development has been extensively demonstrated, the role of BSP in critical illness and sepsis has previously not been elucidated. In contrast, we and others have previously shown an important role of another SIBLING protein, osteopontin (OPN), in critical illness^6,34^, sepsis^35,36^, renal^37^ and heart failure^38^. Like osteopontin, BSP has been shown to be upregulated in renal failure^39^. In contrast, an impaired liver function does not affect BSP serum levels^39^. Interestingly, in our analysis osteopontin and BSP displayed similar effects^6,34,40^: Serum levels of both proteins are elevated in patients with critical illness. Moreover, serum levels of both proteins are correlated to markers of organ function, inflammation and prognosis scores and correlate with an unfavorable prognosis, especially, when elevated at day three after admission to ICU^6^. Thus, it is conceivable that both molecules have a similar functional role in critical disease, which may be most likely be executed via shared functional groups, like the ASARM motif. As stated above, the fibroblast growth factor-23 (FGF23) is regulated by the ASARM motif of SIBLING proteins^30^. FGF23 is a circulating phosphaturic protein that decreases the expression of NPT2, a sodium-phosphate cotransporter in the proximal tubule^41^. Cleavage of intact FGF23 has been demonstrated to be enhanced during inflammation and sepsis in patients with chronic kidney disease^42^. This effect resolves after resolution of infection. It is suggested that this effect might participate into the counter-regulatory response to severe inflammation in critical disease^42^. This effect is likely executed by the shared ASARM motif of SIBLING proteins like osteopontin or BSP^30^. In line with this suggestion, negative regulators of FGF23 like PHEX, which also binds to the ASARM motif, are induced by inflammatory molecules like TNF, which may contribute to hypophosphataemia during sepsis and other inflammatory conditions^43^ causing critical disease. Of note, we could demonstrate a close correlation of BSP serum levels and proinflammatory biomarkers like TNF in our study. BSP serum levels also correlated with the heart failure marker BNP in our collective of critically ill patients. In line, the BSP target FGF23^44^ and the SIBLING protein osteopontin^38^ have been shown to be associated with a poor prognosis of heart failure patients.

Suggesting a role of the BSP/ASARM- PHEX- FGF23 signaling pathway in inflammatory-related hypophosphatemia, our data point to an important issue: The important functional and prognostic role of phosphate imbalance and bone mineralization homeostasis in sepsis and critical disease due to e.g. heart failure or renal failure^45^. Signaling pathways that are involved in bone mineralization seem to be involved into the complex sequence of inflammatory and immunosuppressive stimuli during sepsis and critical disease^46,47^. Further molecular studies are needed to elucidate these complex mechanisms. In addition to the functional importance of the ASARM-PHEX-FGF23 axis, other potential mechanism of action of BSP in sepsis are conceivable: e.g. a direct interaction of BSP with Staphylococcus aureus microbial surface components via Bone Sialoprotein-binding Protein (Bbp)^48^, which may facilitate adherence of the microbes to components of the extracellular matrix of the host.

In summary, our data highlight a potential function of bone sialoprotein in the prognostic judgment of patients during the first days of ICU treatment. Of course, these data need to be confirmed in further longitudinal clinical trials using independent cohorts of before a clinical use can be considered. Finally, our results suggest a previously unrecognized function of BSP in the pathophysiology of critical illness and should trigger further mechanistic research on the role of BSP and SIBLING proteins in general in the regulation of inflammation in this clinical setting.

## Electronic supplementary material

Supplementary information
