# Supplementary material for: Elevated serum levels of bone sialoprotein during ICU treatment predict long-term mortality in critically ill patients
Source: Sci Rep. 2018 Jun 27;8:9750. doi: 10.1038/s41598-018-28201-7 (PMC6021423; doi:10.1038/s41598-018-28201-7)
Supplement: Supplementary file 1 — Supplementary information [file 41598_2018_28201_MOESM1_ESM.pdf]

Elevated serum levels of bone sialoprotein during ICU treatment predict long-term mortality in critically ill patients

Mark Luedde[1,*], Sanchari Roy[2,*], Hans-Joerg Hippe[1,*], David Vargas Cardenas[1], Martina Spehlmann[1], Mihael Vucur[2], Pia Hoening[2], Sven Loosen[2], Norbert Frey[1], Christian Trautwein[2], Tom Luedde[2], Alexander Koch[2,†], Frank Tacke[2,†], Christoph Roderburg[2,†]

Content of the Supplementary information: Supplementary figure 1

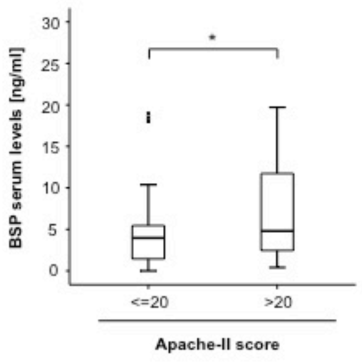

Supplementary Figure 1: BSP concentrations at admission to the ICU were further elevated in patients with more severe disease state according to initial APACHE-II scores (cut-offs are given in the figure).
